# Fish diversity of the largest deltaic formation in the Americas - a description of the fish fauna of the Parnaíba Delta using DNA Barcoding

**DOI:** 10.1038/s41598-019-43930-z

**Published:** 2019-05-17

**Authors:** Aurycéia J. Guimarães-Costa, Fabíola S. Machado, Rory R. S. Oliveira, Vinícius Silva-Costa, Marcelo C. Andrade, Tommaso Giarrizzo, Ulrich Saint-Paul, Iracilda Sampaio, Horacio Schneider

**Affiliations:** 1 Instituto de Ciências Biológicas, Universidade Federal do Pará, Rua Augusto Corrêa, 2-224, Belém, 66077-830 PA Brazil; 2 Instituto de Estudos Costeiros, Universidade Federal do Pará, Alameda Leandro Ribeiro, Bragança, 68600000 PA Brazil; 3 Núcleo de Ecologia Aquática e Pesca da Amazônia, Universidade Federal do Pará, Av. Perimetral 2561, Terra Firme, Belém, 66040-170 PA Brazil; 4 Zentrum für Marine Tropenforschung (ZMT), Fahrenheitstr. 6, 28359 Bremen, Germany

**Keywords:** Biodiversity, Biodiversity, Evolution, Evolutionary biology, Evolutionary biology

## Abstract

Deltas are dynamic and productive systems of enormous ecological significance, encompassing unique and biologically diverse wetland habitats. Here, we present the first data on the molecular diversity of the fish fauna of the Parnaíba Delta, the largest deltaic formation of the Americas. Partial sequences (626 bp) of the mitochondrial *COI* gene (Cytochrome *c* oxidase subunit I) were used to barcode 402 individuals, representing 128 species, belonging to 98 genera, 57 families, 17 orders and two classes. The most abundant orders were the Perciformes, Siluriformes, Gobiiformes, and Pleuronectiformes. The Neighbor-Joining (NJ), Bayesian Inference (BI), and BIN analyses produced 103 molecular clusters, while the Automatic Barcode Gap Discovery (ABGD) and Maximum Likelihood (ML) approaches revealed 102 clusters. The mean conspecific, congeneric and confamilial genetic distances were 0.33%, 14.37%, and 18.60%, respectively. Intraspecific divergence ranged from 0.0% to 1.4%, and all species presented barcode gaps, with the exception of two clusters of *Cathorops spixii* (OTU 96 and OTU 103), which were separated by a low interspecific distance (1.2%), which overlaps the maximum intraspecific genetic distance (1.4%). The barcode data provide new insights into the fish diversity of the Parnaíba Delta, which will be important for the development of further research on this fauna.

## Introduction

River deltas are dynamic and productive systems that have attracted human civilizations around the world for millennia^1^. In most cases, they support high population densities, and are important centers of food production, industry, and economic development. The confluence of fresh and salt waters is also of considerable ecological significance, supporting wetlands with a rich and unique biological diversity^1–4^.

The Parnaíba River Delta (PRD) in northeastern Brazil is considered the largest deltaic formation in the Americas and is the third largest in the world, after the Nile delta in Africa and the Mekong delta in Asia. The Parnaíba Delta encompasses 85 islands within an area of 2,700 km^2^, which includes a variety of ecosystems, such as mangroves, salt marshes, and sandy beaches, that support a rich, but still poorly-known biota^5,6^.

Recent research in the Parnaíba Delta has focused on holocenic geomorphological processes^7^, the bioaccumulation of heavy metals^8^, and morphodynamics and climatic change^9,10^. Over the past 45 years, however, there has been scant research on the morphometric and meristic characters of the local fish assemblage^5,6,11–13^. The fish assemblage of the Parnaíba Delta is composed of freshwater, estuarine and marine species, which face constant fluctuations in conditions, with salinity ranging from freshwater to hypersaline oceanic waters^5,8,9,14^. The inner portion of the Parnaíba Delta, characterized by low salinity levels, is dominated by freshwater species of families such as the Characidae, Cichlidae, and Curimatidae^6,7^. Within the delta, where salinity is higher, there is a predominance of estuarine-marine species, belonging to families such as the Sciaenidae, Carangidae and Ariidae^5,8^. The fish fauna of the Parnaíba Delta also includes sharks and rays (Chondrichthyes), and commercially-important species, such as herrings and tarpons, Actinopterygii^5,14–16^.

In addition to the recognition of species, the comprehensive understanding of fish diversity allows for the more reliable definition of species distributions (in particular, endemism), as well as providing important insights into the ecological characteristics and population density of fish assemblages^17^. The reliable identification of species is fundamental to any study, but can be hampered where species are poorly-known or morphologically similar^18^. In this context, DNA barcoding can provide an accurate molecular diagnosis of species based on the analysis of the sequences of a specific gene^19–21^.

The Cytochrome oxidase I gene (*COI*) is used for the DNA barcoding of fish worldwide, and has proven to be an effective tool for the identification of both adults and larval stages (e.g.^22–30^). The DNA barcoding approach has permitted considerable advances in the understanding of fish diversity, in particular, the elucidation of taxa with similar traits^31–40^. In the present study, the composition of the fish fauna of the Parnaíba Delta was diagnosed by DNA Barcoding, providing new insights into the local fish diversity in this important coastal complex, while also expanding the global barcoding database.

## Results

### Fish diversity

A total of 2,032 fish specimens were collected in the Parnaíba Delta, representing 128 species, 102 of which were recognized by both their morphology and DNA barcoding, and 26 species identified only by morphological cues. The species were distributed in 98 genera, 57 families, 17 orders, and two classes. The 26 species identified only by their morphology were excluded from the molecular analyses due to the poor quality of their DNA (Table S1), with five of these species being identified only to the genus level (*Pomadasys* sp., *Paraclinus* sp., *Phractocephalus* sp., *Steindachneridion* sp., and *Astyanax* sp.). Even though they were identified by both morphological and barcoding analyses, further six taxa (*Poecilia* sp., *Gobiosoma* sp., *Gobionellus* sp., *Hyporhamphus* sp., *Anchoviella* sp., and *Gymnura* sp.) were assigned only to genus because they lack comparable sequences in the BOLD system.

The most abundant orders were the Perciformes (43.8%), Siluriformes (14.1%), Gobiiformes (8.6%), and Pleuronectiformes (5.5%). All other orders contributed less than 3.9% of the total abundance. The Sciaenidae was the most diverse family, with eight species, followed by the Haemulidae and Gobiidae, with seven species each, the Serranidae (6 species), and the Pimelodidae, Gerreidae, and Carangidae, each represented by five species. One of the species recorded here, *Megalops atlanticus*, is classified as vulnerable by the IUCN (International Union for Conservation of Nature), and two others (*Lutjanus analis* and *Lutjanus synagris*) are classified as near-threatened (1.8% of the total), while the vast majority (76.4%) are least concern, although 15.5% of the species have yet to be evaluated.

The Parnaíba Delta provides an important refuge for amphidromous, marine and freshwater fishes. A majority (54.6%) of the species are native to the western Atlantic, while two (1.5%) are endemic of the Parnaíba Delta: *Pterygoplichthys parnaibae* and *Pimelodella parnahybae* (Table S1). All other species inhabit the coastal rivers of South America (10.9%), the eastern and western Atlantic Ocean (8.5%), the western Central Atlantic (5.4%), are circumtropical (4.6%) or are endemic to the southwest Atlantic province (1.5%). Two of the species (*Butis koilomatodon* and *Omobranchus punctatus*) recorded in this study are bioinvaders native to the Indo-Pacific region, which have been introduced unintentionally into the western Atlantic^41,42^, while *Mugil curema* is widely distributed in the Atlantic and eastern Pacific oceans.

### DNA Barcoding: genetic distances and molecular identification

**Figure 1:**
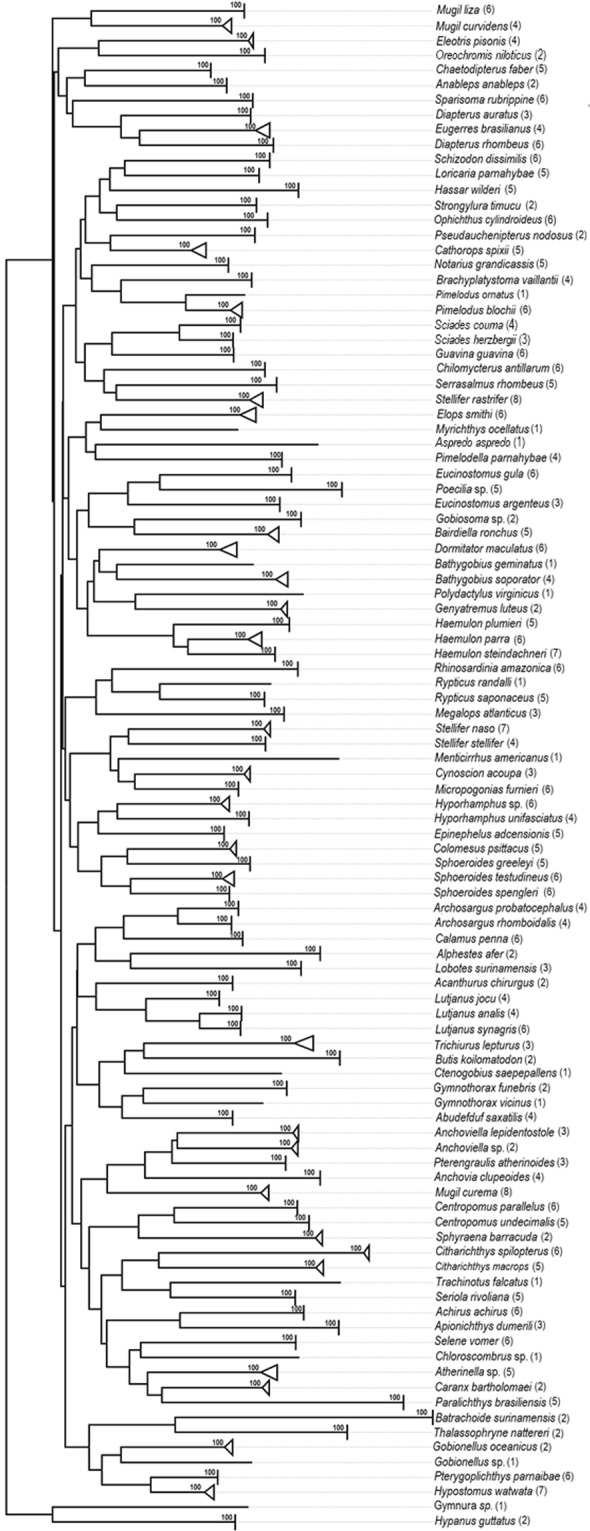
Neighbor-Joining tree based on the fish COI barcodes obtained from the Parnaíba Delta. The values at the nodes are the bootstrap values.

No indels or stop codons were detected in the present analyses. The BIN analyses delimited 103 putative species or OTUs (Table S2). The Neighbour Joining (bootstrap ≥ 97%) and BI topologies indicated the formation of 103 clusters (Fig. 1), whereas the ML and ABGD analyses delimited 102 clusters (P = 0.012915–0.035938). For further details on the composition of the clusters, see Figs S1–S3.

**Figure 2:**
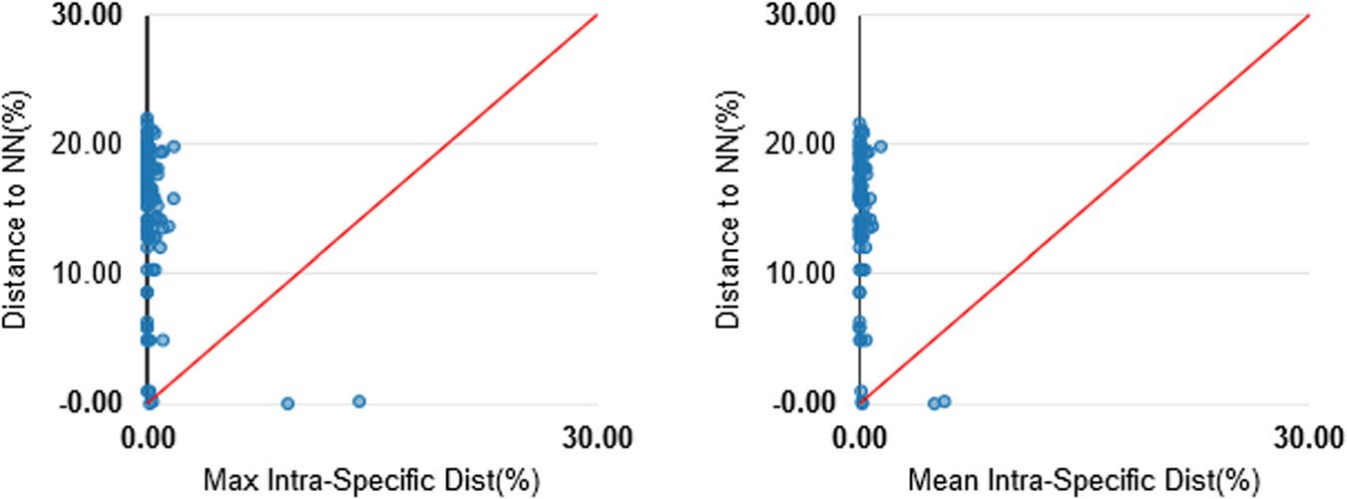
Barcoding gap: Maximum intraspecific Kimura 2-parameter (K2P) distances compared with the minimum interspecific K2P distances recorded in fish from the Parnaíba Delta. The graphs show the overlap of the maximum and mean intra-specific distances with the inter-specific (NN = nearest neighbor) distances.

Mean genetic distances increased with increasing taxonomic level, varying from 0.16 ± 0.00% within species to 14.50 ± 0.01% within genera, and 18.59 ± 0.00% within families (Table 1). Intraspecific distances ranged from 0.0% to 1.4%, while the smallest interspecific distance recorded in the cluster analysis was 1.22%, between *Cathorops spixii* OTU 96 and *C. spixii* OTU 103. The remaining species presented barcode gaps, with a minimum distance between congeners of 4.9%, between *Pimelodus ornatus* and *Pimelodus blochii*, and also *Lutjanus analis* and *Lutjanus synagris*. These distances are much greater than the maximum intraspecific distance, of 1.4% (Fig. 2).

**Table 1:** Summary of the genetic divergence (K2P percentage) at each taxonomic level.

	Min Divergence (%)	Mean Divergence (%)	Max Divergence (%)	SE Divergence (%)
Within Species	0.00	0.16	1.78	0.00
Within Genus	0.96	14.50	21.55	0.01
Within Family	6.33	18.59	31.04	0.00

The NJ topology indicated a lack of monophyly in eight families (Eleotridae, Gobiidae, Sciaenidae, Gerreidae, Carangidae, Ariidae, Loricariidae, and Paralichthyidae). The ML and BI topologies nevertheless recovered monophyletic clusters for all families represented by more than a single genus, except the Gobiidae in the Bayesian Inference (Fig. S2).

## Discussion

### DNA barcoding

This study of the ichthyofauna of the Parnaíba Delta is the first to compile the data in a sequence library, which contributes to the Fish Barcode of Life (FISH-BOL) in the Barcode of Life Data (BOLD) Systems. Relationships among the species are shown in the topology of the NJ tree, in which each terminal node represents an OTU.

The mean intraspecific K2P distance recorded in the present study (0.33%) is similar to those found in marine and freshwater fish species in a number of previous DNA barcoding studies of fish^18,23,25,29,43,44^, *i.e*., 0.3–1.1%, but much higher than the mean value(0.18%) recorded for the fish of the China Sea by^45^.

We analyzed the performance of the DNA barcoding approach for species delimitation using a single locus (*COI*), which is only reliable if there is a sufficient gap between the intraspecific and interspecific divergence, known as the “barcode gap”^46,47^. The BIN analysis found a maximum intraspecific distance of 1.40%, in *Trichiurus lepturus*, while the minimum interspecific distance was 1.22%, recorded between *Cathorops spixii* (OTU 96) and *Cathorops spixii* (OTU 103). There is thus no absolute gap between the maximum intraspecific distance and the minimum interspecific distance if *C. spixii* is considered to be two distinct clusters. However, mean genetic distances between congeners are three and a half times greater than those between individuals of the same species when *C. spixii* is excluded from the analyses, given that the next largest interspecific distance is 4.9%, recorded in two cases, i.e., *Pimelodus ornatus vs*. *Pimelodus blochii* and *Lutjanus analis vs*. *Lutjanus synagris*. In this case, a clear barcode gap exists, which indicates the *COI* was reliable for the precise delimitation of the species of the Parnaíba Delta, in particular for the groups that are difficult to diagnose due to their considerable morphological similarities, *e.g*., sciaenids, *Pimelodus*, and *Loricaria*.

Much lower interspecific genetic distances were found in pairs of fish taxa from the South China Sea^18^, *i.e*., *Gerres oyena vs*. *Gerres japonicus* (0.16%), *Istiblennius edentulous vs*. *Istiblennius lineatus* (0%), and *Uranoscopus oligolepis vs*. *Uranoscopus kaianus* (0.95%), and these findings were attributed to a possible recent process of sympatric speciation. In the present study, a similar situation may account for the closely-related *C. spixii* clusters identified in the BIN analysis, although this can only be confirmed through a more detailed analysis of the population-level differentiation between the two clusters.

The DNA barcoding approach is used primarily to identify taxa and their phylogenetic relationships^36,37,39,48,49^. In the present study, however, six taxa (*Poecilia*, *Gobiosoma*, *Gobionellus*, *Anchoviella*, *Atherinella*, and *Gymnura*) were identified only to the genus level, and this resolution could not be improved using morphological parameters. This limitation suggests: (1) difficulties associated with morphological changes during to the early life history stages of the species in question or (2) the possible existence of new, as yet undescribed species, although any such inference should be treated with caution, in particular because the lack of a positive identification may be at least partly due to the lack of reference sequences in the online database^28^.

### Diversity and endemism

Spatial patterns in the fish assemblage of the Parnaiba Delta varied considerably. The upper areas of the PRD were characterized predominantly by freshwater fish orders such as the Characiformes, Siluriformes and Cichliformes (see^6,11,13^), while the lower PRD and shoreline were dominated by coastal (marine-estuarine) species, whose geographical distributions are not restricted to the Brazilian Province (*sensu*^50^), *i.e*., between the mouth of the Amazon River and the state of Santa Catarina, but range throughout the western Atlantic Ocean. Interestingly, some species with coastal behavior that enter the PRD also inhabit adjacent ecosystems such as oceanic islands and Atlantic coral reefs [e.g. *Gymnothorax funebris* Ranzani, 1839, *Haemulon plumierii* (Lacepède, 1801), *Abudefduf saxa*tilis (Linnaeus 1758), *Sparisoma rubripinne* (Valenciennes, 1840), *Chaetodipterus faber* (Broussonet, 1782), *Sphyraena barracuda* (Edwards)]^51^. The composition of this ichthyofauna was dominated by Perciformes, and sciaenids (8 spp.) and haemulids (7 spp.) were the richest families, including marine species associated with coral reefs. We nevertheless recorded some typical freshwater species that occasionally reach the outer part of the PRD, such as *Astyanax* sp. (Characidae), *Loricaria parnahybae* Steindachner, 1907 and *Hypostomus watwata* Hancock, 1828 (Loricariidae), including the stingray *Potamotrygon orbignyi* (Castelnau)^52^, which is widely distributed in the river systems of the Amazon, Tocantins, Araguaia, and Orinoco basins.

The freshwater habitat has higher levels of species richness and endemism than the marine habitats^53,54^. While Ramos and colleagues^13^ reported 146 species of freshwater fish in the Parnaíba Basin, including 54 endemic species, Guzzi and colleagues^52^ recorded 88, although we recorded 128 species in the delta region alone, including two endemic species. *Pterygoplichthys parnaibae* (Loricariidae) and *Pimelodella parnahybae* (Heptapteridae) are part of the endemic fauna of the hydrographic region between the states of Maranhão and Piauí, which includes the Parnaíba River. *Pterygoplichthys parnaibae* is a strictly freshwater species, which inhabits the upper Parnaiba River^13^. However, recent studies have found the two species in the lower course near the Parnaguá Lagoon and the Poti River^11^, but the present study is the first to report the two species from the Parnaíba Delta. We provide molecular evidence of the existence of possible new species in the PRD. It is important to improve the understanding of the diversity of the fish assemblage in the largest deltaic formation of the Americas, in particular because this is a unique environment, which is still relatively poorly-studied in comparison with other Brazilian estuaries (see^55^).

### Conservation status

Nursery habitats within delta systems provide hydrological connectivity between adjacent ecosystems, contributing to migration and the recruitment of estuarine-dependent species^51^. The Parnaíba Delta not only supports both the freshwater and coastal ichthyofauna, but also species from oceanic regions and coral reefs that are vulnerable to extinction, such as the Atlantic tarpon *Megalops atlanticus* (Megalopidae), and the near-threatened snappers *Lutjanus analis* and *Lutjanus synagris* (Lutjanidae)^56,57^. This area is part of the Delta do Parnaíba Marine Extractive Reserve, which covers an area about 276 km^2 ^^58^ and promotes the sustainable management of artisanal fishing in this region. Such initiatives are important mainly because these fish (tarpon and snapper) are targeted by commercial fisheries, and many other species are being overfished. The findings of the present study thus represent an important first step toward the implementation of effective measures for the conservation and sustainable use of the region’s biodiversity and aquatic resources.

## Methods

### Ethics statement

Fieldwork in the Parnaíba Delta was conducted in accordance with the requirements of the current Brazilian environmental legislation, and was authorized by the Chico Mendes Institute for Biodiversity Conservation (ICMBio), through license number 44679-1 (SISBIO system: Brazilian Environment Ministry). Immediately after collection in the field, the fish specimens were anesthetized with clove oil, fixed in 10% formaldehyde and then preserved in 70% ethanol. These procedures are in accordance with Brazilian federal law 11,974, and were authorized by the Ethics Committee of the Federal University of Pará. All procedures also followed the guidelines of the American Society of Ichthyologists and Herpetologists (ASIH) (http://www.asih.org/pubs/).

### Study Area

The source of the Parnaíba River is located in the Chapada das Mangabeiras, in central Brazil. The river flows 1,716 km north to the Atlantic Ocean, through a catchment area of 322,823 km^2^. The Parnaíba is the longest river contained entirely within Brazil’s Northeast region, and its delta divides the northeastern states of Maranhão and Piauí, and has a shoreline of 30 km on the Atlantic coast of northern South America. The mouth of the delta is divided into five main channels, known as the Parnaíba, Igaraçu, Canárias, Melancieiras, Cajú, and Tutóia rivers. The region has a semi-arid climate, with low annual rainfall (400–800 mm). Salinity ranges from 0 (in the river zone) to 35 (adjacent to the Atlantic Ocean), and the mean annual temperature of the water is 28 °C. The delta is dominated by extensive tracts of mangrove forest, in addition to salt marsh, mudflats and, to a lesser extent, seagrass beds and flat reefs^5,7^.

### Collection and identification of fish specimens

**Figure 3:**
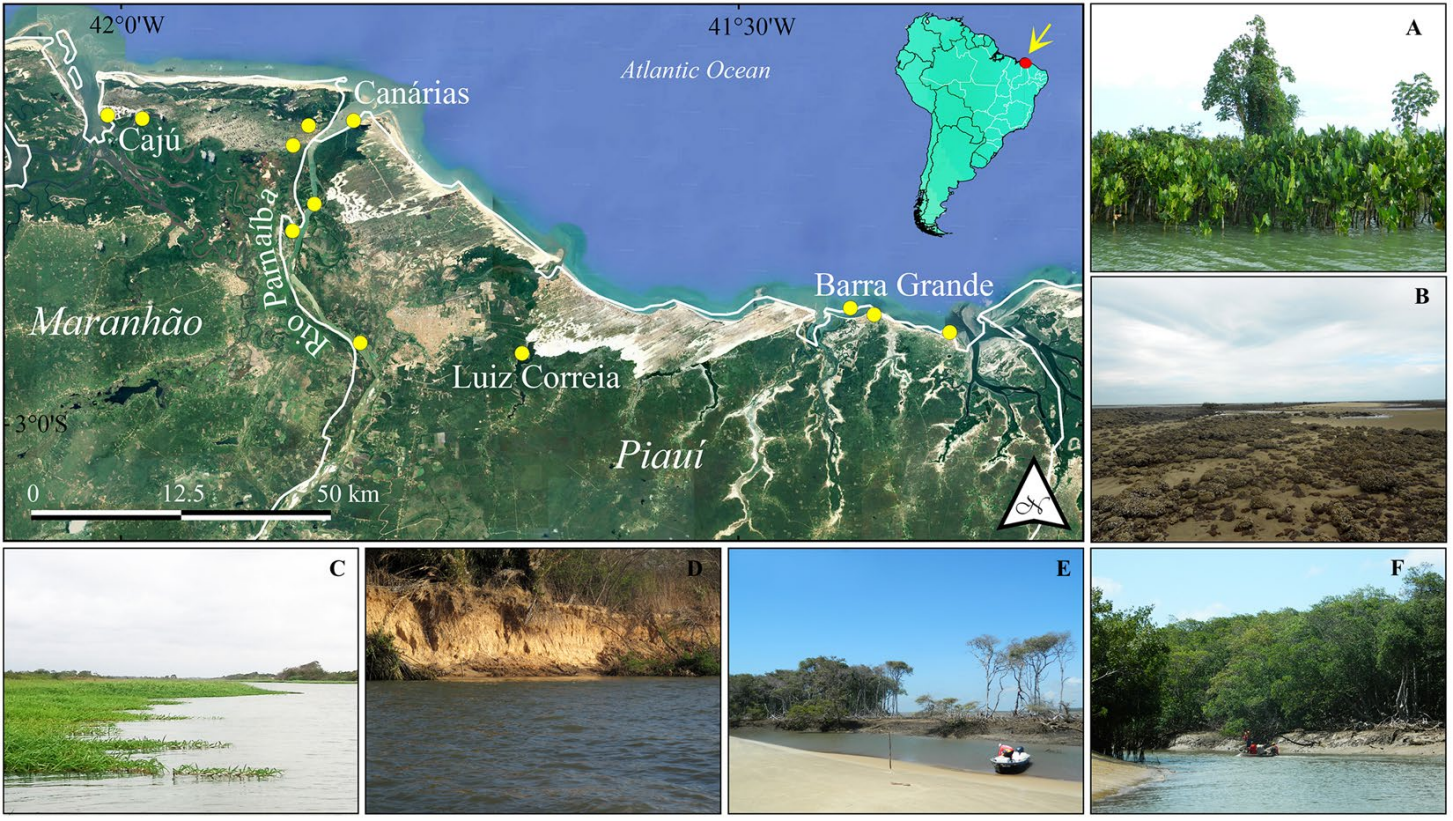
Study area, the Parnaíba Delta, in northeastern Brazil. The yellow dots indicate the fish sampling sites. Landscapes: (**A**) Aquatic macrophytes in Araioses, (**B**) Barra Grande, (**C**) Grass banking in Araioses, (**D**) Confluence with the Iguaraçú River, and (**E**) Channels near Canárias Islands. Map created using QGIS 3.4.0 (Geographic Information System. Open Source Geospatial Foundation) software (https://qgis.org/en/site/).

Specimens were collected in November, 2014, March and April, 2015, and in December, 2015, at 12 points within the Parnaíba Delta (Fig. 3). Samples were taken using a variety of net types, with the objective of maximizing specimen collection in the different habitats of the study area. These nets included bag-shaped seine nets without wings (3 m width, 1.3 m high, with a 3 m bag, and 10 mm mesh) in unvegetated intertidal areas, an otter trawl (7.6 m long, with a 10.43 m footrope and 8.62 m headrope, and a 13 mm mesh in the cod-end) in the main channel, block nets (10 m × 3 m, with a 12 mm mesh) in tidal creeks, and small hand-nets (50 cm in diameter, 2 mm mesh) in diverse aquatic environments, such as mangrove prop roots and tidepools. Specimens were identified to the lowest possible taxonomic level based on the relevant published species descriptions (e.g.^59,60^). A small fragment of muscle tissue was taken from between one and eight specimens per species and stored in 96% ethanol for the barcode analyses. The voucher specimens were fixed in a 10% formalin solution in the field, and then stored in 70% ethanol in the laboratory, where they were deposited in the fish collection of the Aquatic Ecology Group (GEA) at the Federal University of Pará (the GEA catalog is available in Table S3). All the material is available to interested researchers on request.

### Extraction, amplification and sequencing of the DNA

The DNA was extracted using a Wizard Genomic DNA Purification Kit (Promega Corporation, Madison, WI – USA), following the manufacturer’s protocol. A 626 bp fragment of the mitochondrial *COI* gene region was amplified using the primers *COI* 5′ TCAACCAACCACAAAGACATTGGCAC 3′ and *COI* 5′ TAGACTTCTGGGTGGCCAAAGAATCA 3′. The samples were amplified in a final volume of 25 μL, containing 4 μl of DNTP (1.25 mM), 2.5 μl of 10X buffer solution, 1 μl of MgCl2 (25 Mm), 0.25 μl of each primer (200 ng/μl), 1–1.5 μl of genomic DNA (100 ng/μl), 1 U of Taq DNA polymerase (5 U/μl), and purified water to complete the final reaction volume.

The Polymerase Chain Reactions (PCRs) were run in a thermocycler (Applied Biosystems) under the following thermal protocol: initial denaturation at 93 °C for 3 min; 35 cycles of denaturation at 94 °C for 30 s, annealing (at temperatures of 50–60 °C, depending on the species) for 45 s, and extension at 72 °C for 45 s, with a final extension of 5 min at 72 °C. All positive reactions were sequenced in an ABI 3500 automatic sequencer (Applied Biosystems).

### Analysis of the DNA

A database was compiled in Bioedit 7.0.9^61^ from the sequences of 402 specimens. The chromatograms were checked visually before the alignment of the sequences in the Muscle program^62^. The data on the specimens (collection details, accession numbers, and sequence trace files) were implemented in Bold Systems, and the resulting alignment was analyzed phylogenetically using the Neighbour-Joining (NJ) method, with Maximum-Likelihood (ML) and Bayesian Inference (BI) approaches. The NJ tree and the genetic divergences within and between species, genera, and families were formulated using the analytical tools available in the BOLD Systems platform, based on the Kimura 2-Parameter model^63^.

Species were delimited using i) a Barcode Index Numbers (BIN) analysis^64^, based on the uncorrected *p* distances, which provide a single BIN for each Operational Taxonomic Unit (OTU), and ii) the Automatic Barcode Gap Discovery method^47^, which is based on the identification of the “barcode gap”, that is, the gap between the intra- and interspecific pairwise distances recovered from the *COI* sequences. This analysis permits the identification of groups (~species) when the maximum intraspecific distance and the minimum interspecific distance do not overlap. The analysis was run in the online interface http://wwwabi.snv.jussieu.fr/public/abgd/abgdweb.html, and was based on the K2P distance matrix in the MEGA format, which has an X value (relative gap width) of 1.2, and interspecific divergence of 0.001–0.1. The maximum intraspecific divergence was plotted against the minimum interspecific divergence.

The Bayesian Inference (BI) and Maximum Likelihood (ML) analyses were run in the MrBayes 3.1.2^65^ and RAxML 7.2.7^66^ programs, respectively. The evolutionary models were selected in PartitionFinder 1.0.1^67^ for each codon position of the *COI* gene alignment. For MrBayes, the models chosen for each position of the codon were: 1st position of the codon – SYM + G; 2nd position – F81 + G e 3rd position – GTR + I + G. The Bayesian Inference included four chains and two independent runs of 10 million generations. The trees were saved every 10 generations, and 25% of the first trees were discarded as burn-in. The performance of the run and effective sample sizes (ESS >200) were displayed in Tracer 1.5^68^. The ML analysis was run using the GTRGAMMA model for all the partitions established in Partitionfinder, and the confidence of the branches of the best tree was analyzed in detail based on a rapid analysis of 500 bootstrap pseudoreplicates. Each analysis was run independently three times, and in all cases, the results were congruent. The topologies were viewed and edited in FigTree, version 1.4. 3^69^.

## Supplementary information

Supplementary Information

## Data Availability

All the COI sequences of the individuals processed in this study are available in the Bold Systems. The accession codes are available in the Supplementary Information: Figs S1–S3, and Tables S2 and S3.
