# Supplementary material for: Fish diversity of the largest deltaic formation in the Americas - a description of the fish fauna of the Parnaíba Delta using DNA Barcoding
Source: Sci Rep. 2019 May 17;9:7530. doi: 10.1038/s41598-019-43930-z (PMC6525184; doi:10.1038/s41598-019-43930-z)
Supplement: Supplementary file 1 — Supplementary Information [file 41598_2019_43930_MOESM1_ESM.pdf]

Scientific Reports

Fish diversity of the largest deltaic formation in the Americas - a description of the fish fauna of the Parnaíba Delta using DNA Barcoding

Aurycéia J. Guimarães-Costa, Fabíola S. Machado, Rory R. S. Oliveira, Vinícius Silva-Costa, Marcelo Andrade, Tommaso Giarrizzo, Ulrich Saint-Paul, Iracilda Sampaio and Horacio Schneider

*Correspondence:
Dr. Aurycéia J. Guimarães-Costa
auryceia@yahoo.com.br

Supplementary Information

Figure Legend

Figure S1. Topology of the Neighbour-Joining generated for the COI gene sequences of the fish specimens collected in the Parnaíba Delta, northern Brazil. The numbers at the nodes correspond to bootstrap values.

Figure S2. Topology of the Bayesian Inference generated for the COI gene sequences of the fish specimens collected in the Parnaíba Delta, northern Brazil. The numbers at the nodes correspond to posterior probabilities.

Figure S3. Topology of the Maximum Likelihood generated for the COI gene sequences of the fish specimens collected in the Parnaíba Delta, northern Brazil. The numbers at the nodes correspond to bootstrap values.

Figure S1

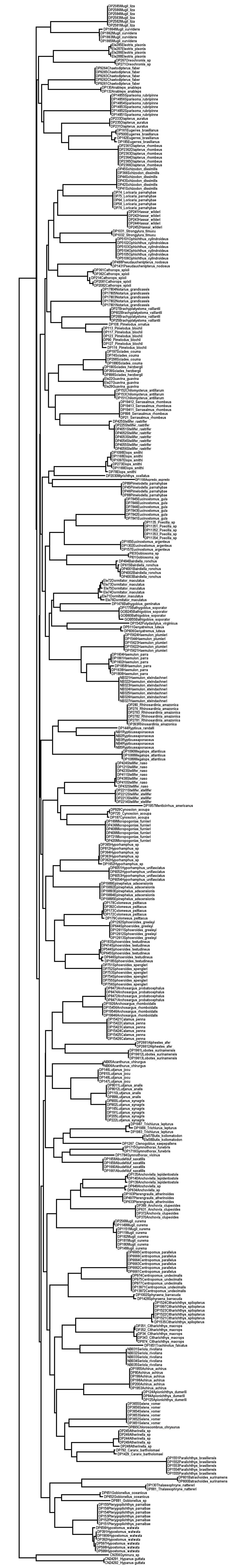

Figure S2

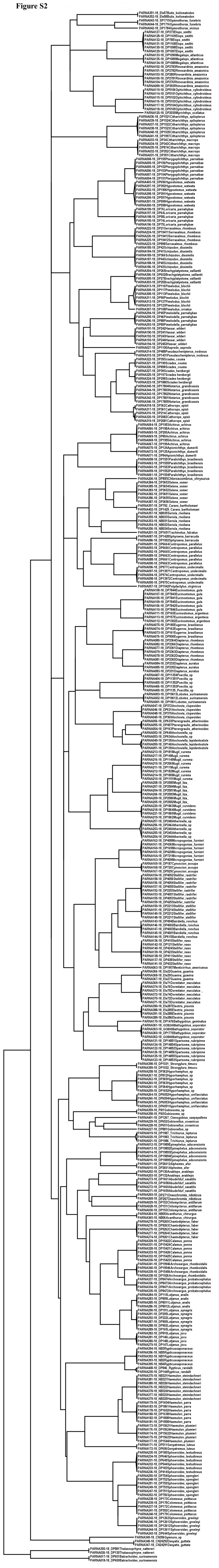

Figure S3

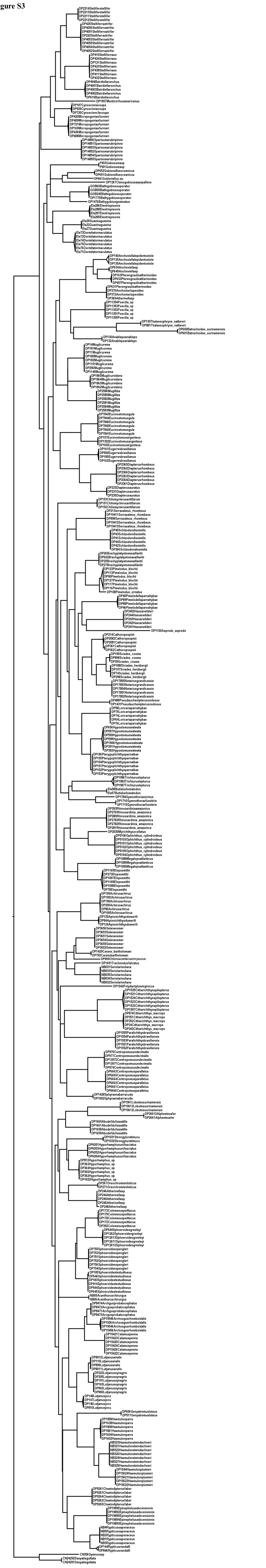

Table 1. Taxonomic classification of the fish species from the Parnaíba Delta identified in the present study. IUCN categories: **NE**= Not Evaluated, **DD** = Data Deficient, **LC** = Least Concern, **NT** = Near Threatened, **VU** = Vulnerable. Geographic range: **WA** = Western Atlantic, **EA** = Eastern Atlantic, **WEA** = Western and Eastern Atlantic, **CT** = Circumtropical, **SWA** = Southwest Atlantic, **WCA** = Western Central Atlantic, **EP** = Eastern Pacific, **IWP** = Indo-Western Pacific, **SA** = South America, **PDR** = Parnaíba Delta River.

Order	Family	Species	Geographic range	Status IUCN	Metodology
**Myliobatiformes**	Dasyatidae	*Hypanus guttatus* (Bloch & Schneider, 1801)	WA	DD	Morfology + Barcode
	Potamotrygonidae	*Potamotrygon orbignyi* (Castelnau, 1855)	Coastal rivers of SA	LC	Morfology
	Gymnuridae	*Gymnura* sp.	-	-	Morfology + Barcode
**Elopiformes**	Elopidae	*Elops smithi* McBride, Rocha, Ruiz-Carus & Bowen, 2010	WA	DD	Morfology + Barcode
	Megalopidae	*Megalops atlanticus* Valenciennes, 1847	WA	VU	Morfology + Barcode
**Anguilliformes**	Muraenidae	*Gymnothorax vicinus* (Castelnau, 1855)	WA	LC	Morfology + Barcode
		Gymnothorax funebris Ranzani, 1839	WA	LC	Morfology + Barcode
	Ophichthidae	*Ophichthus cylindroideus* (Ranzani, 1839)	WA	LC	Morfology + Barcode
		Ahlia egmontis (Jordan, 1884)	WA	LC	Morfology
		Myrichthys ocellatus (Lesueur, 1825)	WA	LC	Morfology + Barcode
**Clupeiformes**	Clupeidae	*Rhinosardinia amazonica* (Steindachner, 1879)	WCA	LC	Morfology + Barcode
	Engraulidae	*Pterengraulis atherinoides* (Linnaeus, 1766)	WA	LC	Morfology + Barcode
		Anchovia clupeoides (Swainson, 1839)	WA	LC	Morfology + Barcode

		Anchoviella lepidentostole (Fowler, 1911)	WA	LC	Morfology + Barcode
		Anchoviella sp.	-	-	Morfology + Barcode
Perciforme	Sciaenidae	*Stellifer naso* (Jordan, 1889)	WA	LC	Morfology + Barcode
		Bairdiella ronchus (Cuvier, 1830)	WA	LC	Morfology + Barcode
		Stellifer stellifer (Bloch, 1790)	WA	DD	Morfology + Barcode
		Stellifer rastrifer (Jordan, 1889)	WA	LC	Morfology + Barcode
		Micropogonias furnieri (Desmarest, 1823)	WA	LC	Morfology + Barcode
		Cynoscion acoupa (Lacepède, 1801)	WA	LC	Morfology + Barcode
		Plagioscion squamosissimus (Heckel, 1840)	WA	-	Morfology
		Menticirrhus americanus (Linnaeus, 1758)	WA	LC	Morfology + Barcode
	Carangidae	*Chloroscombrus chrysurus* (Linnaeus, 1766)	WEA	-	Morfology + Barcode
		Caranx bartholomaei Cuvier, 1833	WEA	LC	Morfology + Barcode
		Trachinotus falcatus (Linnaeus, 1758)	WA	LC	Morfology + Barcode
		Seriola rivoliana Valenciennes, 1833	CT	LC	Morfology + Barcode
		Selene vomer (Linnaeus, 1758)	WA	LC	Morfology + Barcode
	Sparidae	*Calamus penna* (Valenciennes, 1830)	WA	LC	Morfology + Barcode
		Archosargus probatocephalus (Walbaum, 1792)	WA	LC	Morfology + Barcode
		Archosargus rhomboidalis (Linnaeus, 1758)	WA	LC	Morfology + Barcode

	Gerreidae	*Eugerres brasilianus* (Cuvier, 1830)	WA	LC	Morfology + Barcode
		Diapterus rhombeus (Cuvier, 1829)	WA	LC	Morfology + Barcode
		Diapterus auratus Ranzani, 1842	WA	LC	Morfology + Barcode
		Eucinostomus gula (Quoy & Gaimard, 1824)	WA	LC	Morfology + Barcode
		Eucinostomus argenteus Baird & Girard, 1855	WA	LC	Morfology + Barcode
	Haemulidae	*Haemulon plumierii* (Lacepède, 1801)	WA	LC	Morfology + Barcode
		Haemulon parra (Desmarest, 1823)	WA	LC	Morfology + Barcode
		Genyatremus luteus (Bloch, 1790)	WA	-	Morfology + Barcode
		Haemulon steindachneri (Jordan & Gilbert, 1882)	WA	LC	Morfology + Barcode
		Polydactylus virginicus (Linnaeus, 1758)	WA	LC	Morfology + Barcode
		Pomadasys sp.	-	-	Morfology
		Haemulon chrysargyreum Günther, 1859	WA	LC	Morfology
	Lutjanidae	*Lutjanus jocu* (Bloch & Schneider, 1801)	WEA	DD	Morfology + Barcode
		Lutjanus analis (Cuvier, 1828)	WA	NT	Morfology + Barcode
		Lutjanus synagris (Linnaeus, 1758)	WA	NT	Morfology + Barcode
		Lutjanus alexandrei (Moura & Lindeman, 2007)	SWA	NE	Morfology
	Serranidae	*Alphestes afer* (Bloch, 1793)	WA	LC	Morfology + Barcode
		Rypticus saponaceus (Bloch & Schneider, 1801)	WEA	LC	Morfology + Barcode

		Rypticus bistrispinus (Mitchill, 1818)	WA	LC	Morfology
		Rypticus randalli Courtenay, 1967	WA	LC	Morfology + Barcode
		Mycteroperca tigris (Valenciennes, 1833)	WA	LC	Morfology
		Epinephelus adscensionis (Osbeck, 1765)	WEA	LC	Morfology + Barcode
	Centropomidae	*Centropomus parallelus* Poey, 1860	WA	LC	Morfology + Barcode
		Centropomus pectinatus Poey, 1860	WA	LC	Morfology
		Centropomus undecimalis (Bloch, 1792)	WA	LC	Morfology + Barcode
	Lobotidae	*Lobotes surinamensis* (Bloch, 1790)	CT	LC	Morfology + Barcode
	Trichiuridae	*Trichiurus lepturus* Linnaeus, 1758	CT	LC	Morfology + Barcode
	Sphyraenidae	*Sphyraena* sp.	-	-	Morfology
		Sphyraena barracuda (Edwards, 1771)	CT	LC	Morfology + Barcode
	Scaridae	*Sparisoma rubripinne* (Valenciennes, 1840)	WA	LC	Morfology + Barcode
	Cichlidae	*Oreochromis niloticus* (Linnaeus, 1758)	Native from Africa - introduced for aquaculture	-	Morfology + Barcode
	Pomacentridae	*Abudefduf saxatilis* (Linnaeus, 1758)	WEA	LC	Morfology + Barcode
	Ephippidae	*Chaetodipterus faber* (Broussonet, 1782)	WA	LC	Morfology + Barcode
	Acanthuridae	*Acanthurus chirurgus* (Bloch, 1787)	WEA	LC	Morfology + Barcode
	Labrisomidae	*Paraclinus* sp.	-	-	Morfology

	Bleniidae	*Scartella cristata* (Linnaeus, 1758)	CT	LC	Morfology
		Omobranchus punctatus (Valenciennes, 1836)	Native from the IWP - introduced into the WA	LC	Morfology
	Echeneidae	*Remora remora* (Linnaeus, 1758)	CT	LC	Morfology
	Stromateidae	*Peprilus paru* (Linnaeus, 1758)	WA	LC	Morfology
	Chaenopsidae	*Emblemariopsis signifer* (Ginsburg, 1942)	WA	LC	Morfology
**Pleuronectiformes**	Paralichthyidae	*Paralichthys brasiliensis* (Ranzani, 1842)	SWA	NE	Morfology + Barcode
		Citharichthys macrops Dresel, 1885	WA	-	Morfology + Barcode
		Syacium micrurum Ranzani, 1842	WA	LC	Morfology
		Citharichthys spilopterus Günther, 1862	WA	LC	Morfology + Barcode
	Achiridae	*Achirus achirus* (Linnaeus, 1758)	WCA	LC	Morfology + Barcode
		Trinectes paulistanus (Miranda Ribeiro, 1915)	WA	LC	Morfology
	Soleidae	*Apionichthys dumerili* Kaup, 1858	WCA	LC	Morfology + Barcode
**Tetraodontiformes**	Tetraodontidae	*Sphoeroides testudineus* (Linnaeus, 1758)	WA	LC	Morfology + Barcode
		Colomesus psittacus (Bloch & Schneider, 1801)	WA	LC	Morfology + Barcode
		Sphoeroides greeleyi Gilbert, 1900	WA	LC	Morfology + Barcode
		Sphoeroides spengleri (Bloch, 1785)	WEA	LC	Morfology + Barcode
	Diodontidae	*Chilomycterus antillarum* Jordan & Rutter, 1897	WA	LC	Morfology + Barcode

**Mugiliformes**	Mugilidae	*Mugil liza* Valenciennes, 1836	WA	DD	Morfology + Barcode
		Mugil curema Valenciennes, 1836	WA and EP	LC	Morfology + Barcode
		Mugil curvidens Valenciennes, 1836	WEA	NE	Morfology + Barcode
**Gobiiformes**	Gobiidae	*Gobionellus* sp.	-	-	Morfology + Barcode
		Gobionellus oceanicus (Pallas, 1770)	WA	LC	Morfology + Barcode
		Bathygobius geminatus Tornabene, Baldwin & Pezold, 2010	WA	DD	Morfology + Barcode
		Ctenogobius stigmaticus (Poey, 1860)	WA	LC	Morfology
		Ctenogobius saepepallens (Gilbert & Randall, 1968)	WA	LC	Morfology + Barcode
		Gobiosoma sp.	-	-	Morfology + Barcode
		Bathygobius soporator (Valenciennes, 1837)	WEA	LC	Morfology + Barcode
	Eleotridae	*Guavina guavina* (Valenciennes, 1837)	WA	LC	Morfology + Barcode
		Dormitator maculatus (Bloch, 1792)	WA	LC	Morfology + Barcode
		Eleotris pisonis (Gmelin, 1789)	WA	LC	Morfology + Barcode
	Butidae	*Butis koilomatodon* (Bleeker, 1849)	Native from the IWP - introduced into the WA	NE	Morfology + Barcode
**Siluriformes**	Loricariidae	*Loricaria parnahybae* Steindachner, 1907	Coastal rivers of SA	NE	Morfology + Barcode
		Pterygoplichthys parnaibae (Weber, 1991)	PRB	NE	Morfology + Barcode
		Hypostomus watwata Hancock, 1828	Coastal rivers of SA	NE	Morfology + Barcode

	Ariidae	*Cathorops spixii* (Agassiz, 1829) (OTU 96)	WA	NE	Morfology + Barcode
		Cathorops spixii (Agassiz, 1829) (OTU 103)	WA	NE	Morfology + Barcode
		Sciades couma (Valenciennes, 1840)	Coastal rivers of SA	LC	Morfology + Barcode
		Sciades herzbergii (Bloch, 1794)	Coastal rivers of S A and Caribbean	LC	Morfology + Barcode
		Notarius grandicassis (Valenciennes, 1840)	WCA	LC	Morfology + Barcode
	Pimelodidae	*Brachyplatystoma vaillantii* (Valenciennes, 1840)	Coastal rivers of SA	NE	Morfology + Barcode
		Pimelodus blochii Valenciennes, 1840	Coastal rivers of SA	NE	Morfology + Barcode
		Pimelodus ornatus Kner, 1858	Coastal rivers of SA	-	Morfology + Barcode
		Phractocephalus sp.	-	-	Morfology
		Steindachneridion sp.	-	-	Morfology
	Auchenipteridae	*Pseudauchenipterus nodosus* (Bloch, 1794)	Coastal rivers of SA	NE	Morfology + Barcode
		Auchenipterus nuchalis (Spix & Agassiz, 1829)	Coastal rivers of SA	LC	Morfology
	Heptapteridae	*Pimelodella parnahybae* Fowler, 1941	PRB	LC	Morfology + Barcode
	Aspredinidae	*Aspredo aspredo* (Linnaeus, 1758)	WCA	NE	Morfology
	Doradidae	*Hassar wilderi* Kindle, 1895	Coastal rivers of SA	NE	Morfology + Barcode
**Batrachoidiformes**	Batrachoididae	*Thalassophryne nattereri* Steindachner, 1876	WA	LC	Morfology + Barcode
		Batrachoides surinamensis (Bloch & Schneider, 1801)	WCA	LC	Morfology + Barcode

**Characiformes**	Anostomidae	*Schizodon dissimilis* (Garman, 1890)	Coastal rivers of SA	NE	Morfology + Barcode
	Serrasalmidae	*Serrasalmus rhombeus* (Linnaeus, 1766)	Coastal rivers of SA	NE	Morfology + Barcode
	Characidae	*Astyanax sp.*	-	-	Morfology
**Cyprinodontiformes**	Poeciliidae	*Poecilia* sp.	-	-	Morfology + Barcode
	Anablepidae	*Anableps anableps* (Linnaeus, 1758)	Coastal rivers of SA	NE	Morfology + Barcode
**Beloniformes**	Hemiramphidae	*Hyporhamphus* sp.	-	-	Morfology + Barcode
		Hyporhamphus unifasciatus (Ranzani, 1841)	WA	LC	Morfology + Barcode
	Belonidae	*Strongylura timucu* (Walbaum, 1792)	WA	LC	Morfology + Barcode
**Atheriniformes**	Atherinopsidae	*Atherinella brasiliensis* (Quoy & Gaimard, 1825)	WCA	LC	Morfology + Barcode
**Scorpaeniformes**	Dactylopteridae	*Dactylopterus volitans* (Linnaeus, 1758)	WEA	LC	Morfology
**Syngnathiformes**	Syngnathidae	*Anarchopterus tectus* (Dawson, 1978)	WA	LC	Morfology

Table S2. Results of BINs from the "BIN Discordance Report" using BOLD.

Taxa	BIN	BIN Total Members
Citharichthys sp.	BOLD:ADL5719	5
Gobionellus	BOLD:ADL5866	1
Hyporhamphus sp.	BOLD:ADL6070	6
Pimelodus blochii	BOLD:ADL6111	6
Gobiosoma sp.	BOLD:ADL6656	2
Cathorops spixii	BOLD:ADL6931	2
Pimelodus ornatus	BOLD:ADL7324	1
Batrachoides	BOLD:ADL7499	2
Thalassophryne nattereri	BOLD:ADL7500	2
Anchoviella sp.	BOLD:ADL7515	2
Anchovia	BOLD:ADL7516	4
Ophichthus cylindroideus	BOLD:ADL7576	6
Loricaria sp.	BOLD:ADL8521	5
Rhinosardinia	BOLD:ADL8572	6
Gymnura sp.	BOLD:ADL9087	1
Eucinostomus argenteus	BOLD:AAA4224	125
Lutjanus analis	BOLD:AAA5055	80
Lutjanus jocu	BOLD:AAA5843	76
Sphyraena barracuda	BOLD:AAA6100	116
Oreochromis niloticus	BOLD:AAA6537	280
Haemulon parra	BOLD:AAA6815	60
Bathygobius soporator	BOLD:AAA7195	146
Abudefduf saxatilis	BOLD:AAA7275	89
Mugil curema	BOLD:AAA7840	38
Lutjanus synagris	BOLD:AAA9301	127
Trichiurus lepturus	BOLD:AAB0166	26
Acanthurus chirurgus	BOLD:AAB0262	65
Caranx bartholomaei	BOLD:AAB0584	213
Sphoeroides testudineus	BOLD:AAB1130	303
Eucinostomus gula	BOLD:AAB1827	24
Menticirrhus americanus	BOLD:AAB5198	32
Archosargus probatocephalus	BOLD:AAB6459	51

Micropogonias furnieri	BOLD:AAB8513	42
Chaetodipterus faber	BOLD:AAB8805	110
Megalops atlanticus	BOLD:AAB9313	30
Elops smithi	BOLD:AAB9386	97
Seriola rivoliana	BOLD:AAB9420	56
Trachinotus falcatus	BOLD:AAB9494	41
Sphoeroides spengleri	BOLD:AAC0145	21
Poecilia sp.	BOLD:AAC0279	49
Archosargus rhomboidalis	BOLD:AAC0711	29
Ctenogobius saepepallens	BOLD:AAC1502	25
Dormitator maculatus	BOLD:AAC2209	24
Eugerres brasilianus	BOLD:AAC2575	13
Eleotris pisonis	BOLD:AAC5439	19
Haemulon steindachneri	BOLD:AAC5791	18
Gobionellus oceanicus	BOLD:AAC6238	7
Centropomus undecimalis	BOLD:AAC7122	32
Serrasalmus rhombeus	BOLD:AAC7587	319
Rypticus saponaceus	BOLD:AAD3334	30
Rypticus randalli	BOLD:AAD3335	11
Polydactylus virginicus	BOLD:AAD3370	18
Chloroscombrus chrysurus	BOLD:AAD5127	36
Strongylura timucu	BOLD:AAE2157	13
Chilomycterus antillarum	BOLD:AAE2265	52
Citharichthys spilopterus	BOLD:AAE2823	43
Haemulon plumierii	BOLD:AAE3001	12
Centropomus parallelus	BOLD:AAE4140	41
Pterengraulis atherinoides	BOLD:AAE8469	12
Stellifer stellifer	BOLD:AAE8989	10
Selene vomer	BOLD:AAF1235	40
Bathygobius geminatus	BOLD:AAF4010	56
Diapterus rhombeus	BOLD:AAF5627	44
Guavina guavina	BOLD:AAF7351	10
Gymnothorax vicinus	BOLD:AAG1984	21
Myrichthys ocellatus	BOLD:AAH3388	11

Pseudauchenipterus nodosus	BOLD:AAH8177	20
Gymnothorax funebris	BOLD:AAI6444	13
Hyporhamphus unifasciatus	BOLD:AAI7799	7
Cynoscion acoupa	BOLD:AAI9474	9
Stellifer rastrifer	BOLD:AAJ4924	23
Atherinella sp.	BOLD:AAN5135	8
Diapterus auratus	BOLD:AAO1630	9
Cathorops spixii	BOLD:AAU0758	15
Notarius grandicassis	BOLD:AAU2011	18
Calamus penna	BOLD:AAW8346	8
Sphoeroides greeleyi	BOLD:AAZ0868	54
Anchoviella lepidentostole	BOLD:AAZ7817	20
Dasyatis guttata	BOLD:ABW1681	3
Colomesus psittacus	BOLD:ACA6880	52
Mugil curvidens	BOLD:ACC0101	22
Butis koilomatodon	BOLD:ACE0185	7
Bairdiella ronchus	BOLD:ACE3340	9
Mugil liza	BOLD:ACE4593	225
Schizodon dissimilis	BOLD:ACL3191	32
Apionichthys dumerili	BOLD:ACM8566	4
Achirus achirus	BOLD:ACM8577	42
Aspredo aspredo	BOLD:ACQ9923	4
Sciades sp.	BOLD:ACR8600	9
Sciades couma	BOLD:ACR8601	12
Hassar wilderi	BOLD:ACR8662	21
Stellifer naso	BOLD:ACS2557	11
Sparisoma rubripinne	BOLD:ACU0159	17
Paralichthys brasiliensis	BOLD:ACU4991	6
Brachyplatystoma vaillantii	BOLD:ACZ9033	11
Anableps anableps	BOLD:ADC4239	12
Pimelodella parnahybae	BOLD:ADC4518	16
Hypostomus watwata	BOLD:ADL5854	11

Tabela S3. Taxon, sample ID, species and museum ID for specimens analysed in the study.

Taxon	Sampleid	Species	Museum ID
Lobotidae	DP1961Lobotes_surinamensis	*Lobotes surinamensis*	GEA 07592
Lobotidae	DP19612Lobotes_surinamensis_	*Lobotes surinamensis*	GEA 07592
Lobotidae	DP19613Lobotes_surinamensis_	*Lobotes surinamensis*	GEA 07592
Loricariidae	DP151Pterygoplichthys_parnaibae	*Pterygoplichthys parnaibae*	*
Loricariidae	DP152Pterygoplichthys_parnaibae_	*Pterygoplichthys parnaibae*	*
Loricariidae	DP153Pterygoplichthys_parnaibae_	*Pterygoplichthys parnaibae*	*
Loricariidae	DP154Pterygoplichthys_parnaibae_	*Pterygoplichthys parnaibae*	*
Loricariidae	DP155Pterygoplichthys_parnaibae_	*Pterygoplichthys parnaibae*	*
Loricariidae	DP156Pterygoplichthys_parnaibae_	*Pterygoplichthys parnaibae*	*
Serranidae	DP2661Alphestes_afer	*Alphestes afer*	*
Serranidae	DP26612Alphestes_afer_	*Alphestes afer*	*
Serranidae	DP1989Epinephelus_adscensionis	*Epinephelus adscensionis*	GEA 04277
Serranidae	DP19892Epinephelus_adscensionis_	*Epinephelus adscensionis*	GEA 04277
Serranidae	DP19893Epinephelus_adscensionis_	*Epinephelus adscensionis*	GEA 04277
Serranidae	DP19894Epinephelus_adscensionis_	*Epinephelus adscensionis*	GEA 04277
Serranidae	DP19895Epinephelus_adscensionis_	*Epinephelus adscensionis*	GEA 04277
Batrachoididae	DP601Batrachoides surinamensis	*Batrachoides surinamensis*	GEA 04412
Batrachoididae	DP600Batrachoides surinamensis	*Batrachoides surinamensis*	GEA 04824
Trichiuridae	DP1987_Trichiurus_lepturus	*Trichiurus lepturus*	GEA 04233
Trichiuridae	DP1983_Trichiurus_lepturus	*Trichiurus lepturus*	GEA 04215
Trichiuridae	DP1986_Trichiurus_lepturus	*Trichiurus lepturus*	GEA 04257
Batrachoididae	DP130Thalassophryne_nattereri	*Thalassophryne nattereri*	GEA 04263
Batrachoididae	DP881Thalassophryne_nattereri	*Thalassophryne nattereri*	GEA 04261
Aspredinidae	DP1100Aspredo_aspredo	*Aspredo aspredo*	GEA 05257
Diodontidae	DP151Chilomycterus_antillarum_	*Chilomycterus antillarum*	GEA 07525
Diodontidae	DP152Chilomycterus_antillarum_	*Chilomycterus antillarum*	GEA 07525
Diodontidae	DP153Chilomycterus_antillarum_	*Chilomycterus antillarum*	GEA 07525
Paralichthyidae	DP351Citharichthys_sp_	*Citharichthys macrops*	GEA 04576
Paralichthyidae	DP352Citharichthys_sp_	*Citharichthys macrops*	GEA 07472
Paralichthyidae	DP34Citharichthys	*Citharichthys macrops*	GEA 04576

Paralichthyidae	DP343Citharichthys_	*Citharichthys macrops*	GEA 04607
Paralichthyidae	DP874Citharichthys_	*Citharichthys macrops*	GEA 05273
Paralichthyidae	DP1521Citharichthys_spilopterus_	*Citharichthys spilopterus*	GEA 05265
Paralichthyidae	DP1522Citharichthys_spilopterus_	*Citharichthys spilopterus*	GEA 05265
Paralichthyidae	DP1523Citharichthys_spilopterus_	*Citharichthys spilopterus*	GEA 05265
Paralichthyidae	DP1524Citharichthys_spilopterus_	*Citharichthys spilopterus*	GEA 05265
Paralichthyidae	DP1535Citharichthys_spilopterus_	*Citharichthys spilopterus*	GEA 04424
Paralichthyidae	DP1997Citharichthys_spilopterus	*Citharichthys spilopterus*	GEA 04424
Muraenidae	DP1784Gymnothorax_vicinus	*Gymnothorax vicinus*	GEA 07589
Muraenidae	DP1715Gymnothorax_funebris	*Gymnothorax funebris*	GEA 04210
Muraenidae	DP1716Gymnothorax_funebris	*Gymnothorax funebris*	GEA 04259
Engraulidae	DP369Anchovia_clupeoides	*Anchovia clupeoides*	GEA 04952
Engraulidae	DP631Anchovia_clupeoides	*Anchovia clupeoides*	GEA 04493
Engraulidae	DP372Anchovia_clupeoides	*Anchovia clupeoides*	GEA 07548
Engraulidae	DP370Anchovia_clupeoides	*Anchovia clupeoides*	GEA 07548
Engraulidae	DP135Anchoviella_lepidentostole	*Anchoviella lepidentostole*	GEA 04369
Engraulidae	DP140Anchoviella_lepidentostole	*Anchoviella lepidentostole*	GEA 04461
Engraulidae	DP139Anchoviella_lepidentostole	*Anchoviella lepidentostole*	GEA 04462
Engraulidae	DP163Pterengraulis_atherinoides	*Pterengraulis atherinoides*	GEA 02540
Engraulidae	DP497Pterengraulis_atherinoides	*Pterengraulis atherinoides*	GEA 02512
Engraulidae	DP633Pterengraulis_atherinoides	*Pterengraulis atherinoides*	GEA 05124
Engraulidae	DP649Anchoviella_sp	*Anchoviella sp.*	GEA 05226
Engraulidae	DP634Anchoviella_sp	*Anchoviella sp.*	GEA 05090
Paralichthyidae	DP1551Paralichthys_brasiliensis_	*Paralichthys_brasiliensis*	GEA 04768
Paralichthyidae	DP1552Paralichthys_brasiliensis_	*Paralichthys_brasiliensis*	GEA 04768
Paralichthyidae	DP1553Paralichthys_brasiliensis_	*Paralichthys_brasiliensis*	GEA 04768
Paralichthyidae	DP1554Paralichthys_brasiliensis_	*Paralichthys_brasiliensis*	GEA 04768
Paralichthyidae	DP1555Paralichthys_brasiliensis_	*Paralichthys_brasiliensis*	GEA 04768
Polynemidae	DP1542Polydactylus_virginicus	*Polydactylus virginicus*	GEA 07634
Achiridae	DP1953Achirus_achirus	*Achirus achirus*	GEA 04481
Achiridae	DP200Achirus_achirus	*Achirus achirus*	GEA 04373
Achiridae	DP198Achirus_achirus	*Achirus achirus*	GEA 04483
Achiridae	DP199Achirus_achirus	*Achirus achirus*	GEA 04469
Achiridae	DP1955Achirus_achirus	*Achirus achirus*	GEA 04203

Achiridae	DP80Achirus_achirus	*Achirus achirus*	GEA 04374
Sciaenidae	DP1957Menticirrhus_americanus	*Menticirrhus americanus*	GEA 04231
Gerreidae	DP161Eugerres_brasilianus	*Eugerres brasilianus*	GEA 04619
Gerreidae	DP162Eugerres_brasilianus	*Eugerres brasilianus*	GEA 04596
Gerreidae	DP185Eugerres_brasilianus	*Eugerres brasilianus*	GEA 04612
Gerreidae	DP500Eugerres_brasilianus	*Eugerres brasilianus*	GEA 04822
Soleidae	DP124Apionichthys_dumerili	*Apionichthys dumerili*	GEA 04806
Soleidae	DP84Apionichthys_dumerili	*Apionichthys dumerili*	GEA 05259
Soleidae	DP125Apionichthys_dumerili	*Apionichthys dumerili*	GEA 05350
Gerreidae	DP231Diapterus_auratus	*Diapterus auratus*	GEA 05276
Gerreidae	DP233Diapterus_auratus	*Diapterus auratus*	GEA 05275
Gerreidae	DP235Diapterus_auratus	*Diapterus auratus*	GEA 05272
Gerreidae	DP2361Diapterus_rhombeus_	*Diapterus rhombeus*	GEA 05270
Gerreidae	DP2362Diapterus_rhombeus_	*Diapterus rhombeus*	GEA 05270
Gerreidae	DP2363Diapterus_rhombeus_	*Diapterus rhombeus*	GEA 05270
Gerreidae	DP2364Diapterus_rhombeus_	*Diapterus rhombeus*	GEA 05270
Gerreidae	DP2365Diapterus_rhombeus_	*Diapterus rhombeus*	GEA 05270
Gerreidae	DP2366Diapterus_rhombeus_	*Diapterus rhombeus*	GEA 05270
Centropomidae	DP6661Centropomus_parallelus_	*Centropomus parallelus*	GEA 05034
Centropomidae	DP6662Centropomus_parallelus_	*Centropomus parallelus*	GEA 05034
Centropomidae	DP6663Centropomus_parallelus_	*Centropomus parallelus*	GEA 05034
Centropomidae	DP6664Centropomus_parallelus_	*Centropomus parallelus*	GEA 05034
Centropomidae	DP6665Centropomus_parallelus_	*Centropomus parallelus*	GEA 05034
Centropomidae	DP6666Centropomus_parallelus_	*Centropomus parallelus*	GEA 05034
Sphyraenidae	DP1002Sphyraena_barracuda	*Sphyraena barracuda*	GEA 04823
Sphyraenidae	DP1428Sphyraena_barracuda	*Sphyraena barracuda*	GEA 04823
Clupeidae	DP393Rhinosardinia_amazonica	*Rhinosardinia amazonica*	GEA 04978
Clupeidae	DP2781Rhinosardinia_amazonica	*Rhinosardinia amazonica*	GEA 05133
Clupeidae	DP2782Rhinosardinia_amazonica	*Rhinosardinia amazonica*	GEA 05100
Clupeidae	DP2783Rhinosardinia_amazonica	*Rhinosardinia amazonica*	GEA 05135
Clupeidae	DP280Rhinosardinia_amazonica	*Rhinosardinia amazonica*	GEA 05136
Clupeidae	DP276Rhinosardinia_amazonica	*Rhinosardinia amazonica*	GEA 04597
Centropomidae	DP974Centropomus_undecimalis	*Centropomus undecimalis*	GEA 04605
Centropomidae	DP975Centropomus_undecimalis	*Centropomus undecimalis*	GEA 02510

Centropomidae	DP977Centropomus_undecimalis	*Centropomus undecimalis*	GEA 04427
Centropomidae	DP13971Centropomus_undecimalis_	*Centropomus undecimalis*	GEA 04807
Centropomidae	DP13972Centropomus_undecimalis_	*Centropomus undecimalis*	GEA 04807
Gerreidae	DP165Eucinostomus_argenteus	*Eucinostomus argenteus*	GEA 04621
Gerreidae	DP1302Eucinostomus_argenteus	*Eucinostomus argenteus*	GEA 04621
Gerreidae	DP157Eucinostomus_argenteus	*Eucinostomus argenteus*	GEA 04621
Gerreidae	DP7841Eucinostomus_gula_	*Eucinostomus gula*	GEA 07576
Gerreidae	DP7842Eucinostomus_gula_	*Eucinostomus gula*	GEA 07576
Gerreidae	DP7843Eucinostomus_gula_	*Eucinostomus gula*	GEA 07576
Gerreidae	DP7844Eucinostomus_gula_	*Eucinostomus gula*	GEA 07576
Gerreidae	DP7845Eucinostomus_gula_	*Eucinostomus gula*	GEA 07576
Gerreidae	DP7846Eucinostomus_gula_	*Eucinostomus gula*	GEA 07576
Scaridae	DP14851Sparisoma_rubripinne_	*Sparisoma rubripinne*	GEA 07521
Scaridae	DP14852Sparisoma_rubripinne_	*Sparisoma rubripinne*	GEA 04995
Scaridae	DP14853Sparisoma_rubripinne_	*Sparisoma rubripinne*	GEA 07522
Scaridae	DP14854Sparisoma_rubripinne_	*Sparisoma rubripinne*	GEA 07522
Scaridae	DP14855Sparisoma_rubripinne_	*Sparisoma rubripinne*	GEA 07522
Scaridae	DP14856Sparisoma_rubripinne_	*Sparisoma rubripinne*	GEA 07522
Elopidae	DP273Elops_smithi	*Elops smithi*	GEA 07481
Elopidae	DP1169Elops_smithi	*Elops smithi*	GEA 05354
Elopidae	DP1097Elops_smithi	*Elops smithi*	GEA 05366
Elopidae	DP1098Elops_smithi	*Elops smithi*	GEA 07439
Elopidae	DP1168Elops_smithi	*Elops smithi*	GEA 07378
Megalopidae	DP78Elops_smithi	*Elops smithi*	GEA 05198
Ophichthidae	DP5101Ophichthus_sp_	*Ophichthus cylindroideus*	GEA 02490
Ophichthidae	DP5102Ophichthus_sp_	*Ophichthus cylindroideus*	GEA 02490
Ophichthidae	DP5103Ophichthus_sp_	*Ophichthus cylindroideus*	GEA 02490
Ophichthidae	DP5104Ophichthus_sp_	*Ophichthus cylindroideus*	GEA 02490
Ophichthidae	DP5105Ophichthus_sp_	*Ophichthus cylindroideus*	GEA 02490
Ophichthidae	DP5106Ophichthus_sp_	*Ophichthus cylindroideus*	GEA 02490
Ophichthidae	DP2030Myrichthys_ocellatus	*Myrichthys ocellatus*	GEA 04196
Sciaenidae	DP494Bairdiella_ronchus	*Bairdiella ronchus*	GEA 04752
Sciaenidae	DP615Bairdiella_ronchus	*Bairdiella ronchus*	GEA 02506
Sciaenidae	DP4001Bairdiella_ronchus_	*Bairdiella ronchus*	GEA 05113

Sciaenidae	DP4002Bairdiella_ronchus_	*Bairdiella ronchus*	GEA 02496
Sciaenidae	DP4003Bairdiella_ronchus_	*Bairdiella ronchus*	GEA 05087
Sciaenidae	DP2211Stellifer_stellifer_	*Stellifer stellifer*	GEA 04758
Sciaenidae	DP2212Stellifer_stellifer_	*Stellifer stellifer*	GEA 04760
Sciaenidae	DP2213Stellifer_stellifer_	*Stellifer stellifer*	GEA 04757
Sciaenidae	DP2214Stellifer_stellifer_	*Stellifer stellifer*	GEA 04759
Megalopidae	DP1090Megalops_atlanticus	*Megalops atlanticus*	GEA 05341
Megalopidae	DP1088Megalops_atlanticus	*Megalops atlanticus*	GEA 05342
Megalopidae	DP1089Megalops_atlanticus	*Megalops atlanticus*	GEA 05340
Sciaenidae	DP410Stellifer_naso	*Stellifer naso*	GEA 03779
Sciaenidae	DP438Stellifer_naso	*Stellifer naso*	GEA 04639
Sciaenidae	DP411Stellifer_naso	*Stellifer naso*	GEA 04463
Sciaenidae	DP423Stellifer_naso	*Stellifer naso*	GEA 04464
Sciaenidae	DP424Stellifer_naso	*Stellifer naso*	GEA 04365
Sciaenidae	DP432Stellifer_naso	*Stellifer naso*	GEA 04646
Sciaenidae	DP121Stellifer_naso	*Stellifer naso*	GEA 04645
Sciaenidae	DP929Cynoscion_acoupa	*Cynoscion acoupa*	GEA 04473
Sciaenidae	DP720Cynoscion_acoupa	*Cynoscion acoupa*	GEA 04473
Sciaenidae	DP187Cynoscion_acoupa	*Cynoscion acoupa*	GEA 04367
Gobiidae	DP1476Bathygobius_geminatus	*Bathygobius geminatus*	GEA 07529
Sciaenidae	DP225Stellifer_rastrifer	*Stellifer rastrifer*	GEA 04578
Sciaenidae	DP4051Stellifer_rastrifer_	*Stellifer rastrifer*	GEA 04761
Sciaenidae	DP4052Stellifer_rastrifer_	*Stellifer rastrifer*	GEA 04755
Sciaenidae	DP4053Stellifer_rastrifer_	*Stellifer rastrifer*	GEA 04763
Sciaenidae	DP4054Stellifer_rastrifer_	*Stellifer rastrifer*	GEA 04578
Sciaenidae	DP4055Stellifer_rastrifer_	*Stellifer rastrifer*	GEA 04761
Sciaenidae	DP4056Stellifer_rastrifer_	*Stellifer rastrifer*	GEA 04760
Sciaenidae	DP425Stellifer_rastrifer	*Stellifer rastrifer*	GEA 04755
Sciaenidae	DP169Micropogonias_furnieri	*Micropogonias furnieri*	GEA 05102
Sciaenidae	DP436Micropogonias_furnieri	*Micropogonias furnieri*	GEA 05102
Sciaenidae	DP408Micropogonias_furnieri	*Micropogonias furnieri*	GEA 05102
Sciaenidae	DP409Micropogonias_furnieri	*Micropogonias furnieri*	GEA 05111
Sciaenidae	DP721Micropogonias_furnieri	*Micropogonias furnieri*	GEA 07536
Sciaenidae	DP420Micropogonias_furnieri	*Micropogonias furnieri*	GEA 07536

Haemulidae	DP1602Haemulon_parra	*Haemulon parra*	GEA 05027
Haemulidae	DP1604Haemulon_parra	*Haemulon parra*	GEA 07519
Haemulidae	DP1638Haemulon_parra	*Haemulon parra*	GEA 04965
Haemulidae	DP1858Haemulon_parra	*Haemulon parra*	GEA 04997
Haemulidae	DP1808Haemulon_parra	*Haemulon parra*	GEA 07520
Haemulidae	DP1861Haemulon_parra	*Haemulon parra*	GEA 04278
Anostomidae	DP41Schizodon_dissimilis_	*Schizodon dissimilis*	GEA 04414
Anostomidae	DP42Schizodon_dissimilis_	*Schizodon dissimilis*	GEA 04364
Anostomidae	DP43Schizodon_dissimilis_	*Schizodon dissimilis*	GEA 04620
Anostomidae	DP44Schizodon_dissimilis_	*Schizodon dissimilis*	GEA 04540
Anostomidae	DP45Schizodon_dissimilis_	*Schizodon dissimilis*	GEA 04467
Anostomidae	DP366Schizodon_dissimilis_	*Schizodon dissimilis*	GEA 04611
Carangidae	DP511Genyatremus_luteus	*Genyatremus luteus*	GEA 05122
Carangidae	DP606Genyatremus_luteus	*Genyatremus luteus*	GEA 07505
Haemulidae	DP15621Haemulon_plumieri_	*Haemulon plumieri*	GEA 04998
Haemulidae	DP15622Haemulon_plumieri_	*Haemulon plumieri*	GEA 04998
Haemulidae	DP15623Haemulon_plumieri_	*Haemulon plumieri*	GEA 04998
Haemulidae	DP15624Haemulon_plumieri_	*Haemulon plumieri*	GEA 04998
Haemulidae	DP1544Haemulon_plumieri	*Haemulon plumieri*	GEA 05029
Carangidae	DP1651Trachinotus_falcatus	*Trachinotus falcatus*	GEA 04410
Carangidae	DP895Chloroscombrus_sp	*Chloroscombrus chrysurus*	GEA. 05323
Loricariidae	DP74Loricaria_sp	*Loricaria parahybae*	GEA 04560
Loricariidae	DP75Loricaria_sp	*Loricaria parahybae*	GEA 04357
Loricariidae	DP64Loricaria_sp	*Loricaria parahybae*	GEA 04341
Loricariidae	DP58Loricaria_sp	*Loricaria parahybae*	GEA 04345
Loricariidae	DP76Loricaria_sp	*Loricaria parahybae*	GEA 04398
Doradidae	DP241Hassar_wilderi_	*Hassar wilderi*	*
Doradidae	DP242Hassar_wilderi_	*Hassar wilderi*	*
Doradidae	DP243Hassar_wilderi_	*Hassar wilderi*	*
Doradidae	DP244Hassar_wilderi_	*Hassar wilderi*	*
Doradidae	DP2452Hassar_wilderi_	*Hassar wilderi*	*
Mugilidae	DP1864Mugil_curvidens	*Mugil curvidens*	GEA 05045
Mugilidae	DP1862Mugil_curvidens	*Mugil curvidens*	GEA 05045
Mugilidae	DP1863Mugil_curvidens	*Mugil curvidens*	GEA 05042

Mugilidae	DP1865Mugil_curvidens	*Mugil curvidens*	GEA 04281
Mugilidae	DP1151Mugil_curema	*Mugil curema*	GEA 04618
Mugilidae	DP11Mugil_curema	*Mugil curema*	GEA 04617
Mugilidae	DP182Mugil_curema	*Mugil curema*	GEA 05044
Mugilidae	DP181Mugil_curema	*Mugil curema*	GEA 04609
Mugilidae	DP180Mugil_curema	*Mugil curema*	GEA 04600
Mugilidae	DP256Mugil_curema	*Mugil curema*	GEA 04370
Mugilidae	DP1149Mugil_curema	*Mugil curema*	GEA 05319
Mugilidae	DP14Mugil_curema	*Mugil curema*	GEA.0441
Mugilidae	DP2581Mugil_liza_	*Mugil liza*	GEA 04490
Mugilidae	DP2582Mugil_liza_	*Mugil liza*	GEA 04278
Mugilidae	DP2583Mugil_liza_	*Mugil liza*	GEA 04278
Mugilidae	DP2584Mugil_liza_	*Mugil liza*	GEA 04278
Mugilidae	DP2585Mugil_liza_	*Mugil liza*	GEA 04278
Mugilidae	DP2586Mugil_liza_	*Mugil liza*	GEA. 04278
Anablepidae	DP130Anableps_anableps	*Anableps anableps*	GEA 03782
Anablepidae	DP132Anableps_anableps	*Anableps anableps*	GEA 03778
Serranidae	DP144Rypticus_randalli	*Rypticus randalli*	GEA 04762
Chordata	DP846_Rypticus_randalli	*Rypticus randalli*	GEA 05206
Gobiidae	DP651Gobionellus_oceanicus	*Gobionellus oceanicus*	GEA 05290
Gobiidae	DP652Gobionellus_oceanicus	*Gobionellus oceanicus*	GEA 05088
Gobiidae	DP881Gobionellus_sp	*Gobionellus sp.*	GEA 05299
Serrasalmidae	DP21Serrasalmus_rhombeus	*Serrasalmus rhombeus*	GEA 04418
Serrasalmidae	DP898Serrasalmus_rhombeus	*Serrasalmus rhombeus*	GEA 07428
Serrasalmidae	DP19411Serrasalmus_rhombeus_	*Serrasalmus rhombeus*	GEA 04811
Serrasalmidae	DP19412Serrasalmus_rhombeus_	*Serrasalmus rhombeus*	GEA 04811
Serrasalmidae	DP19413Serrasalmus_rhombeus_	*Serrasalmus rhombeus*	GEA 04811
Tetraodontidae	DP751Sphoeroides_spengleri_	*Sphoeroides spengleri*	GEA 04174
Tetraodontidae	DP752Sphoeroides_spengleri_	*Sphoeroides spengleri*	GEA 04784
Tetraodontidae	DP753Sphoeroides_spengleri_	*Sphoeroides spengleri*	GEA 04786
Tetraodontidae	DP754Sphoeroides_spengleri_	*Sphoeroides spengleri*	GEA 04171
Tetraodontidae	DP755Sphoeroides_spengleri_	*Sphoeroides spengleri*	GEA 04748
Tetraodontidae	DP756Sphoeroides_spengleri_	*Sphoeroides spengleri*	GEA 04783
Tetraodontidae	DP1262Sphoeroides_greeleyi	*Sphoeroides greeleyi*	GEA 03780

Tetraodontidae	DP644Sphoeroides_greeleyi	*Sphoeroides greeleyi*	GEA 05210
Tetraodontidae	DP12611Sphoeroides_greeleyi_	*Sphoeroides greeleyi*	GEA 05210
Tetraodontidae	DP12612Sphoeroides_greeleyi_	*Sphoeroides greeleyi*	GEA 05210
Tetraodontidae	DP12613Sphoeroides_greeleyi_	*Sphoeroides greeleyi*	GEA 05210
Tetraodontidae	DP172Colomesus_psittacus	*Colomesus psittacus*	GEA 04606
Tetraodontidae	DP173Colomesus_psittacus	*Colomesus psittacus*	GEA 03777
Tetraodontidae	DP178Colomesus_psittacus	*Colomesus psittacus*	GEA 04280
Tetraodontidae	DP179Colomesus_psittacus	*Colomesus psittacus*	GEA 04477
Tetraodontidae	DP392Colomesus_psittacus	*Colomesus psittacus*	GEA 04289
Tetraodontidae	DP645Sphoeroides_testudineus	*Sphoeroides testudineus*	GEA 04488
Tetraodontidae	DP544Sphoeroides_testudineus	*Sphoeroides testudineus*	GEA 05185
Tetraodontidae	DP183Sphoeroides_testudineus	*Sphoeroides testudineus*	GEA 03780
Tetraodontidae	DP646Sphoeroides_testudineus	*Sphoeroides testudineus*	GEA 05231
Tetraodontidae	DP185Sphoeroides_testudineus	*Sphoeroides testudineus*	GEA 04488
Tetraodontidae	DP414Sphoeroides_testudineus	*Sphoeroides testudineus*	GEA 05121
Pomacentridae	DP1658Abudefduf_saxatilis	*Abudefduf saxatilis*	GEA 04247
Pomacentridae	DP1659Abudefduf_saxatilis	*Abudefduf saxatilis*	GEA 07524
Pomacentridae	DP1660Abudefduf_saxatilis	*Abudefduf saxatilis*	GEA 04216
Pomacentridae	DP1661Abudefduf_saxatilis	*Abudefduf saxatilis*	GEA 04254
Ephippidae	DP9261Chaetodipterus_faber_	*Chaetodipterus faber*	GEA 04409
Ephippidae	DP9262Chaetodipterus_faber_	*Chaetodipterus faber*	GEA 04399
Ephippidae	DP9263Chaetodipterus_faber_	*Chaetodipterus faber*	GEA 04403
Ephippidae	DP9264Chaetodipterus_faber_	*Chaetodipterus faber*	GEA 05230
Ephippidae	DP9265Chaetodipterus_faber_	*Chaetodipterus faber*	GEA 05101
Lutjanidae	DP147Lutjanus_jocu	*Lutjanus jocu*	GEA 04598
Lutjanidae	DP148Lutjanus_jocu	*Lutjanus jocu*	GEA 03781
Lutjanidae	DP146Lutjanus_jocu	*Lutjanus jocu*	GEA 04468
Lutjanidae	DP810Lutjanus_jocu	*Lutjanus jocu*	GEA 02544
Lutjanidae	DP899Lutjanus_analis	*Lutjanus analis*	GEA 05301
Lutjanidae	DP110Lutjanus_analis	*Lutjanus analis*	GEA 05301
Lutjanidae	DP8011Lutjanus_analis_	*Lutjanus analis*	GEA 05301
Lutjanidae	DP8012Lutjanus_analis_	*Lutjanus analis*	GEA 05301
Atherinopsidae	DP249Atherinella_sp	*Atherinella sp.*	GEA 05244
Atherinopsidae	DP244Atherinella_sp	*Atherinella sp.*	GEA 05237

Atherinopsidae	DP245Atherinella_sp	*Atherinella sp.*	GEA 05289
Atherinopsidae	DP246Atherinella_sp	*Atherinella sp.*	GEA 07441
Atherinopsidae	DP248Atherinella_sp	*Atherinella sp.*	GEA 05238
Hemiramphidae	DP362Hyporhamphus_sp	*Hyporhamphus sp.*	GEA. 04635
Hemiramphidae	DP1652Hyporhamphus_sp	*Hyporhamphus sp.*	GEA 04635
Hemiramphidae	DP363Hyporhamphus_sp	*Hyporhamphus sp.*	GEA 04635
Hemiramphidae	DP364Hyporhamphus_sp	*Hyporhamphus sp.*	GEA 04635
Hemiramphidae	DP365Hyporhamphus_sp	*Hyporhamphus sp.*	GEA 04635
Hemiramphidae	DP812Hyporhamphus_sp	*Hyporhamphus sp.*	GEA 04635
Hemiramphidae	DP6051Hyporhamphus_unifasciatus_	*Hyporhamphus unifasciatus*	GEA 05250
Hemiramphidae	DP6052Hyporhamphus_unifasciatus_	*Hyporhamphus unifasciatus*	GEA 05250
Hemiramphidae	DP6053Hyporhamphus_unifasciatus_	*Hyporhamphus unifasciatus*	GEA 05250
Hemiramphidae	DP6054Hyporhamphus_unifasciatus_	*Hyporhamphus unifasciatus*	GEA 05250
Cichlidae	DP267Oreochromis_sp	*Oreochromis niloticus*	GEA 07474
Cichlidae	DP271Oreochromis_sp	*Oreochromis niloticus*	GEA 07475
Pimelodidae	DP108Pimelodus_ornatus	*Pimelodus ornatus*	GEA 04383
Pimelodidae	DP113Pimelodus_blochii	*Pimelodus blochii*	GEA 04415
Pimelodidae	DP117Pimelodus_blochii	*Pimelodus blochii*	GEA 04555
Pimelodidae	DP123Pimelodus_blochii	*Pimelodus blochii*	GEA 04360
Pimelodidae	DP90Pimelodus_blochii	*Pimelodus blochii*	GEA 04343
Pimelodidae	DP127Pimelodus_blochii	*Pimelodus blochii*	GEA 04459
Pimelodidae	DP116Pimelodus_blochii	*Pimelodus blochii*	GEA 04454
Auchenipteridae	DP488Pseudauchenipterus_nodosus	*Pseudauchenipterus nodosus*	GEA 07538
Auchenipteridae	DP1431Pseudauchenipterus_nodosus	*Pseudauchenipterus nodosus*	GEA 07541
Lutjanidae	DP900Lutjanus_synagris	*Lutjanus synagris*	GEA 05304
Lutjanidae	DP902Lutjanus_synagris	*Lutjanus synagris*	GEA 05300
Lutjanidae	DP165Lutjanus_synagris	*Lutjanus synagris*	GEA 05305
Lutjanidae	DP191Lutjanus_synagris	*Lutjanus synagris*	GEA 05058
Lutjanidae	DP205Lutjanus_synagris	*Lutjanus synagris*	GEA 02519
Lutjanidae	DP222Lutjanus_synagris	*Lutjanus synagris*	GEA 04832
Heptapteridae	DP89Pimelodella_parnahybae	*Pimelodella parnahybae*	GEA 07478
Heptapteridae	DP45Pimelodella_parnahybae	*Pimelodella parnahybae*	GEA 04342
Heptapteridae	DP46Pimelodella_parnahybae	*Pimelodella parnahybae*	GEA 04438
Heptapteridae	DP88Pimelodella_parnahybae	*Pimelodella parnahybae*	GEA 07466

Loricariidae	DP392Hypostomus_watwata	*Hypostomus watwata*	GEA 07535
Loricariidae	DP391Hypostomus_watwata	*Hypostomus watwata*	GEA 07534
Loricariidae	DP597Hypostomus_watwata	*Hypostomus watwata*	GEA 07534
Loricariidae	DP598Hypostomus_watwata	*Hypostomus watwata*	GEA 07534
Loricariidae	DP599Hypostomus_watwata	*Hypostomus watwata*	GEA 07534
Loricariidae	DP1906Hypostomus_watwata	*Hypostomus watwata*	GEA 07534
Loricariidae	DP656_Hypostomus_sp	*Hypostomus watwata*	GEA 07534
Pimelodidae	DP25Brachyplatystoma_vaillantii	*Brachyplatystoma vaillantii*	GEA 07552
Pimelodidae	DP26Brachyplatystoma_vaillantii	*Brachyplatystoma vaillantii*	GEA 07552
Pimelodidae	DP27Brachyplatystoma_vaillantii	*Brachyplatystoma vaillantii*	GEA. 07552
Pimelodidae	DP602Brachyplatystoma_vaillantii	*Brachyplatystoma vaillantii*	GEA 07552
Ariidae	DP17801Notarius_grandicassis_	*Notarius grandicassis*	GEA 04263
Ariidae	DP17802Notarius_grandicassis_	*Notarius grandicassis*	GEA 04263
Ariidae	DP17803Notarius_grandicassis_	*Notarius grandicassis*	GEA 04263
Ariidae	DP17804Notarius_grandicassis_	*Notarius grandicassis*	GEA 04263
Ariidae	DP17805Notarius_grandicassis_	*Notarius grandicassis*	GEA 04263
Sparidae	DP1926Archosargus_rhomboidalis	*Archosargus rhomboidalis*	GEA 04745
Sparidae	DP15456Archosargus_rhomboidalis	*Archosargus rhomboidalis*	GEA 04744
Sparidae	DP19546Archosargus_rhomboidalis	*Archosargus rhomboidalis*	GEA 04746
Sparidae	DP19946Archosargus_rhomboidalis	*Archosargus rhomboidalis*	GEA 04962
Gymnuridae	CN356Gymnura_sp	*Gymnura sp.*	*
Dasyatidae	CN24291Hypanus_guttatus	*Hypanus guttatus*	*
Dasyatidae	CN24292Hypanus_guttatus	*Hypanus guttatus*	*
Ariidae	DP1880Sciades_couma	*Sciades couma*	GEA 04601
Ariidae	DP298Sciades_couma	*Sciades couma*	GEA 04601
Ariidae	DP197Sciades_couma	*Sciades couma*	GEA 04601
Ariidae	DP74Sciades_couma	*Sciades couma*	GEA 04601
Ariidae	DP190Sciades_herzbergii	*Sciades herzbergii*	GEA 04601
Ariidae	DP39Sciades_herzbergii	*Sciades herzbergii*	GEA 04471
Ariidae	DP898Sciades_herzbergii	*Sciades herzbergii*	GEA 04371
Sparidae	DP8471Archosargus_probatocephalus_	*Archosargus probatocephalus*	GEA 04977
Sparidae	DP8472Archosargus_probatocephalus_	*Archosargus probatocephalus*	GEA 04977
Sparidae	DP8473Archosargus_probatocephalus_	*Archosargus probatocephalus*	GEA 04977
Sparidae	DP8474Archosargus_probatocephalus_	*Archosargus probatocephalus*	GEA 04977

Sparidae	DP15421Calamus_penna_	*Calamus penna*	GEA 07684
Sparidae	DP15422Calamus_penna_	*Calamus penna*	GEA 07684
Sparidae	DP15423Calamus_penna_	*Calamus penna*	GEA 07684
Sparidae	DP15424Calamus_penna_	*Calamus penna*	GEA 07684
Sparidae	DP15425Calamus_penna_	*Calamus penna*	GEA 07684
Sparidae	DP15426Calamus_penna_	*Calamus penna*	GEA 07684
Eleotridae	Ele22Guavina_guavina	*Guavina guavina*	GEA 07537
Eleotridae	Ele27Guavina_guavina	*Guavina guavina*	GEA 07537
Eleotridae	Ele29Guavina_guavina	*Guavina guavina*	GEA 07537
Acanthuridae	NB05Acanthurus_chirurgus	*Acanthurus chirurgus*	GEA 07577
Acanthuridae	NB06Acanthurus_chirurgus	*Acanthurus chirurgus*	GEA 07577
Haemulidae	NB321Haemulon_steindachneri_	*Haemulon steindachneri*	GEA 07578
Haemulidae	NB322Haemulon_steindachneri_	*Haemulon steindachneri*	GEA 07578
Haemulidae	NB323Haemulon_steindachneri_	*Haemulon steindachneri*	GEA 07578
Haemulidae	NB324Haemulon_steindachneri_	*Haemulon steindachneri*	GEA 07578
Haemulidae	NB325Haemulon_steindachneri_	*Haemulon steindachneri*	GEA 07578
Haemulidae	NB326Haemulon_steindachneri_	*Haemulon steindachneri*	GEA 07578
Haemulidae	NB327Haemulon_steindachneri_	*Haemulon steindachneri*	GEA 07578
Eleotridae	Ele71Dormitator_maculatus	*Dormitator maculatus*	GEA 07579
Eleotridae	Ele72Dormitator_maculatus	*Dormitator maculatus*	GEA 07579
Eleotridae	Ele73Dormitator_maculatus	*Dormitator maculatus*	GEA 07579
Eleotridae	Ele74Dormitator_maculatus	*Dormitator maculatus*	GEA 07579
Eleotridae	Ele75Dormitator_maculatus	*Dormitator maculatus*	GEA 07579
Eleotridae	Ele76Dormitator_maculatus	*Dormitator maculatus*	GEA 07579
Carangidae	NB031Seriola_rivoliana_	*Seriola rivoliana*	GEA 07580
Carangidae	NB032Seriola_rivoliana_	*Seriola rivoliana*	GEA 07580
Carangidae	NB033Seriola_rivoliana_	*Seriola rivoliana*	GEA 07580
Carangidae	NB034Seriola_rivoliana_	*Seriola rivoliana*	GEA 07580
Carangidae	NB035Seriola_rivoliana_	*Seriola rivoliana*	GEA 07580
Butidae	Ele57Butis_koilomatodon	*Butis koilomatodon*	GEA 07581
Butidae	Ele58Butis_koilomatodon	*Butis koilomatodon*	GEA 07581
Gobiidae	DP1775Bathygobius_soporator	*Bathygobius soporator*	GEA 05153
Gobiidae	GOB245Bathigobios_soporator	*Bathygobius soporator*	GEA 05225
Gobiidae	GOB60Bathigobios_soporator	*Bathygobius soporator*	GEA 05173

Gobiidae	GOB55Bathigobios_soporator	*Bathygobius soporator*	GEA 04218
Gobiidae	PI03Gobiosoma_sp	*Gobiosoma sp.*	*
Gobiidae	PI01Gobiosoma_sp	*Gobiosoma sp.*	*
Chordata	DP1267_Ctenogobius_saepepallens	*Ctenogobius saepepallens*	*
Ariidae	DP214Cathorops_spixii	*Cathorops spixii*	GEA 04466
Ariidae	DP2081Cathorops_spixii_	*Cathorops spixii*	GEA 04579
Ariidae	DP2082Cathorops_spixii_	*Cathorops spixii*	GEA 04425
Chordata	DP1031_Strongylura_timucu	*Strongylura timucu*	GEA 05284
Chordata	DP1032_Strongylura_timucu	*Strongylura timucu*	GEA 05282
Chordata	DP1135_Poecillia_sp	*Poecillia sp.*	GEA 05329
Chordata	DP11351Poecilia_sp	*Poecillia sp.*	GEA 05334
Chordata	DP11352Poecilia_sp	*Poecillia sp.*	GEA 05312
Chordata	DP11353Poecilia_sp	*Poecillia sp.*	GEA 05322
Chordata	DP11354Poecilia_sp	*Poecillia sp.*	GEA 05330
Serranidae	NB1Rypticussaponaceus	*Rypticus saponaceus*	GEA 07582
Serranidae	NB2Rypticussaponaceus	*Rypticus saponaceus*	GEA 07582
Serranidae	NB3Rypticussaponaceus	*Rypticus saponaceus*	GEA 07582
Serranidae	NB4Rypticussaponaceus	*Rypticus saponaceus*	GEA 07582
Serranidae	NB5Rypticussaponaceus	*Rypticus saponaceus*	GEA 07582
Chordata	DP792_Caranx_bartholomaei	*Caranx bartholomaei*	GEA 04936
Chordata	DP1429_Caranx_bartholomaei	*Caranx bartholomaei*	GEA 04936
Carangidae	DP3651Selene_vomer_	*Selene vomer*	GEA 04288
Carangidae	DP3652Selene_vomer_	*Selene vomer*	GEA 04411
Carangidae	DP3653Selene_vomer_	*Selene vomer*	GEA 04288
Carangidae	DP3654Selene_vomer_	*Selene vomer*	GEA 04411
Carangidae	DP3655Selene_vomer_	*Selene vomer*	GEA 07533
Carangidae	DP3656Selene_vomer_	*Selene vomer*	GEA. 04288
Eleotridae	Ele285Eleotris_pisonis	*Eleotris pisonis*	GEA 07583
Eleotridae	Ele286Eleotris_pisonis	*Eleotris pisonis*	GEA 07583
Eleotridae	Ele287Eleotris_pisonis	*Eleotris pisonis*	GEA 07583
Eleotridae	Ele288Eleotris_pisonis	*Eleotris pisonis*	GEA 07583
Ariidae	DP361Cathorops_spixii_	*Cathorops spixii*	GEA 04371
Ariidae	DP362Cathorops_spixii_	*Cathorops spixii*	GEA 04371

Species identified by morphology

Potamotrygonidae	Potamotrygon_orbignyi	*Potamotrygon orbignyi*	GEA 05247
Ophichthidae	Ahlia_egmontis	*Ahlia egmontis*	GEA 04207
Sciaenidae	Plagioscion_squamosissimus	*Plagioscion squamosissimus*	*
Haemulidae	Pomadasys_sp	*Pomadasys* sp.	GEA 07685
Serranidae	Rypticus_bistrispinus	*Rypticus subbifrenatus*	GEA 02509
Serranidae	Mycteroperca_tigris	*Mycteroperca tigris*	GEA 04273
Centropomidae	Centropomus_pectinatus	*Centropomus pectinatus*	GEA 04813
Labrisomidae	Paraclinus_sp	*Paraclinus* sp.	*
Bleniidae	Scartella_cristata	*Scartella cristata*	GEA 04212
Bleniidae	Omobranchus_punctatus	*Omobranchus punctatus*	GEA 04222
Echeneidae	Remora_remora	*Remora remora*	GEA 04235
Stromateidae	Peprilus_paru	*Peprilus paru*	ICT 07527
Chaenopsidae	Emblemariopsis_signifer	*Emblemariopsis signifer*	*
Paralichthyidae	Syacium_micrurum	*Syacium micrurum*	GEA 04768
Gobiidae	Ctenogobius_stigmaticus	*Ctenogobius stigmaticus*	*
Pimelodidae	Phractocephalus_sp	*Phractocephalus* sp.	*
Pimelodidae	Steindachneridion sp	*Steindachneridion* sp.	GEA 04766
Auchenipteridae	Auchenipterus_nuchalis	*Auchenipterus nuchalis*	GEA 04630
Characidae	Astyanax_sp	*Astyanax* sp.	GEA 05254
Dactylopteridae	Dactylopterus_volitans	*Dactylopterus volitans*	*
Syngnathidae	Anarchopterus_tectus	*Anarchopterus tectus*	GEA 04963

*Samples of fish tissue assigned by local fishermen.
